# Evaluating the remineralisation potential and clinical evidence of emerging biocompatible materials in restorative dentistry: a systematic review

**DOI:** 10.2340/biid.v13.45679

**Published:** 2026-04-15

**Authors:** Duraiarasan Isaiselvi, Kariannan Partheeban Indumathi, Siluvai Sibyl, Govindaraj Krishnaprakash

**Affiliations:** Department of Public Health Dentistry, Faculty of Medicine and Health Sciences, SRM Kattankulathur Dental College and Hospitals, SRM Institute of Science and Technology, Chengalpattu, India

**Keywords:** Aloe vera, chitosan, dental caries, grape seed, herbal, non-fluoridated biocompatible materials, remineralisation, restorative dentistry, self-assembling peptides

## Abstract

**Background:**

Biocompatible materials with remineralising and regenerative properties are now being increasingly used in restorative dentistry as possible materials in place of conventional agents. This systematic review aimed to evaluate the laboratory remineralisation potential and available clinical evidence of emerging biocompatible, non-fluoridated materials used in restorative dentistry. Patient-reported outcomes were explored where reported.

**Materials and methods:**

A systematic review was performed on relevant databases as PubMed, Cochrane, EBSCOhost and Scopus.

**Results:**

A total of 8 in vivo and 23 in vitro studies were included. Self-assembling peptide P11–4 (SAP P11–4) and chitosan-based materials demonstrated consistent remineralisation benefits, predominantly in laboratory studies, with limited supporting clinical evidence. Risk of bias was moderate for in vitro studies and low-to-some concerns for in vivo studies.

**Conclusion:**

Self-assembling peptides and chitosan-based biomaterials show promising potential, though current evidence is largely laboratory-based and requires further clinical validation. Further well-designed clinical trials are warranted.

**KEY MESSAGES:**

- Self-assembling peptide SAP P11–4 demonstrates strong clinical evidence for minimally invasive remineralisation of early carious lesions.
- Chitosan-based restorative and preventive materials provide comparable outcomes to conventional materials with added biocompatibility benefits.
- Although promising, most emerging biocompatible agents require further high-quality clinical trials to validate long-term effectiveness and patient outcomes.

## Introduction

Dental caries is highly preventable, but remains one of the most prevalent chronic diseases in children and adults worldwide [1]. The WHO Global Oral Health Status Report estimated that globally 2 billion people suffer from caries of permanent teeth and 514 million children suffer from caries of primary teeth [2]. Shafer defined dental caries as an ‘irreversible microbial disease of the calcified tissues of the teeth, characterized by demineralization of the inorganic portion and destruction of the organic substance of the tooth, which often leads to cavitation’ [3]. The enamel and dentinal mineral loss are due to the acid attacks produced by the bacterial metabolism of carbohydrates and sugars [4]. Fortunately, dental caries is reversible during the initial phase of the disease, and the stagnation of enamel and dentin demineralisation can be prevented through the inhibition of biofilm formation and salivary protective factors [5]. Restoring a decayed tooth is essential for preventing further deterioration, preserving tooth structure, and maintaining oral function. Untreated decay can impair general health and quality of life by causing pain, infection, and tooth loss. According to the literature search [4, 6] numerous substances including fluorides, calcium glycerophosphate, and xylitol, have been employed to effectively allow demineralised dentin and enamel to remineralise.

Several researchers have used both traditional fluoridated and non-fluoridated dentifrices to examine the demineralisation and remineralisation of enamel lesions in permanent teeth. Though it has advantages, excessive levels of fluoride can be harmful leading to dental and skeletal fluorosis, hypersensitivity reactions, hypersalivation, dyspnoea, stomach irritation, muscular spasm, birth abnormalities, and so on [6, 7]. Therefore, alternative remineralising agents were explored, and consequently, a novel approach to managing enamel and dentin remineralisation has focused on identifying natural, effective, and non-fluoride-based treatment options. Biocompatible materials have been created to overcome these obstacles and enhance patient outcomes and treatment effectiveness. Despite the stability and aesthetic appeal of conventional restoratives like amalgam and composite resins, research into natural alternatives has been prompted by concerns about biocompatibility and durability. This systematic review was conducted to evaluate the laboratory remineralisation potential and available clinical evidence of emerging non-fluoridated biocompatible materials used in restorative dentistry.

## Materials and methods

**Research question:** The research question was framed in PEO format.

In patients undergoing restorative dental procedures, how do emerging biocompatible materials perform in terms of clinical effectiveness, durability, and surrogate clinical outcomes, with patient-reported outcomes evaluated when reported?

**P (Population):** Patients receiving restorative dental treatments.

**E (Exposure):** Emerging biocompatible materials (e.g. herbal based materials like chitosan, cellulose, self-assembling peptides, grapeseed, non-fluoride-based materials).

**O (Outcome):** Surrogate clinical outcomes (lesion regression, surface integrity, remineralisation indices), laboratory outcomes (surface microhardness, SEM/EDX, Ca/P ratio), and patient- reported outcomes when reported.

**Figure 1:**
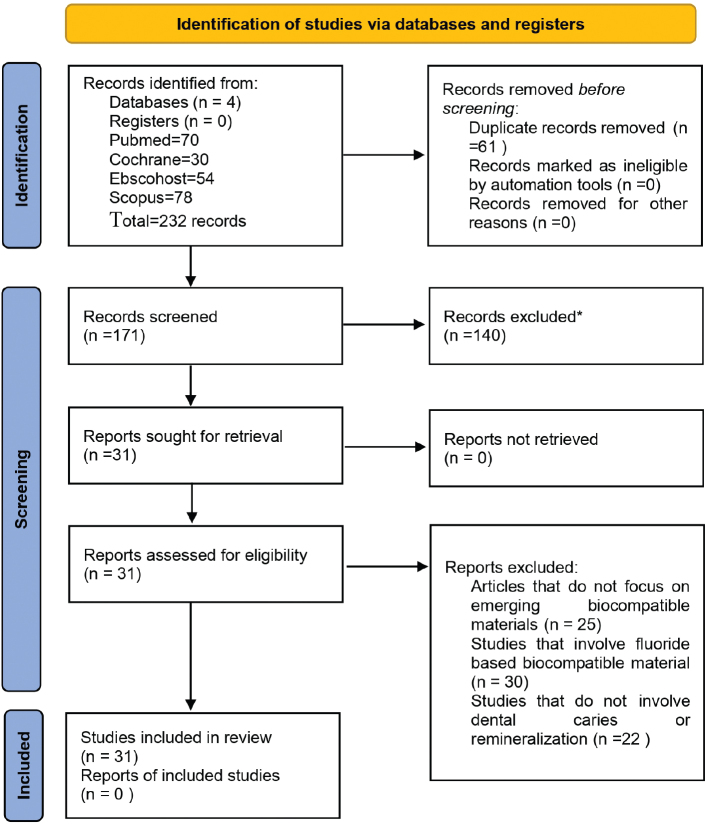
PRISMA 2020 flow diagram illustrating study selection and screening process. Source: Authors’ analysis.

**Protocol and registration:** The protocol for systematic review is registered with PROSPERO (International Prospective Register of Systematic Review) with registration number (CRD420251011817) and follows PRISMA 2020 guidelines. Figure 1 shows the PRISMA flowchart of study selection.

### Inclusion criteria

1. Studies involving patients undergoing restorative dental procedures.
2. Use of emerging biocompatible materials (herbal and non-fluoride based) such as plant-derived biomaterials, self-assembling peptides, chitosan, cellulose, Aloe vera, and grape seed extract in restorative dentistry.
3. Articles available in English.
4. Studies published from 2015 to 2025 to focus on emerging materials.
5. Original studies, including experimental, observational, and clinical trials, together with case–control studies, were selected for review.

### Exclusion criteria

1. Studies evaluating interventions not related to restorative dentistry.
2. Articles published in languages other than English.
3. Studies that focus exclusively on fluoride-based materials.
4. Studies not addressing dental caries or remineralisation.

## Search strategy

A broad search was conducted in databases like PUBMED, COCHRANE, EBSCOhost and SCOPUS using the keywords like plant derived materials, grapeseed, chitosan, Aloe vera, self-assembling peptides, herbal, non-fluoridated materials, remineralising agent, dental caries, remineralisation, and restorative-dentistry. BOOLEAN expression like **AND** and **OR** were used. Duplicates were removed. The search was last updated on 15 March 2025. Two reviewers independently extracted data from included studies; any disagreements were resolved by discussion.

### Data synthesis

A quantitative meta-analysis was not feasible due to heterogeneity in study designs, interventions, and outcome measures among the included studies. Therefore, a narrative synthesis of the results was conducted in accordance with PRISMA 2020 guidelines*.*

### Risk of bias assessment (Methods section)

**Figure 2:**
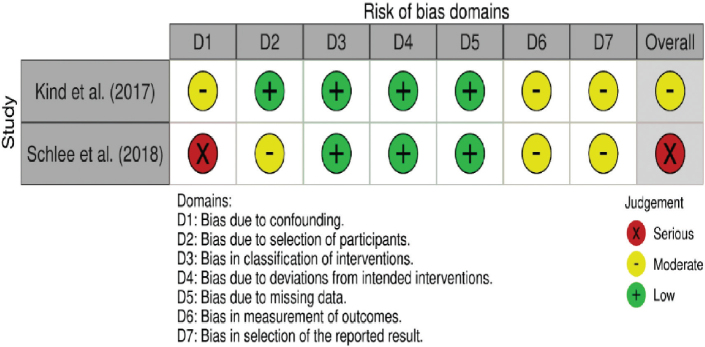
Risk of bias assessment for in vitro studies using Quality in Non- Randomised Studies (QUIN) tool. Source: Authors’ analysis.

Risk of bias for included case series and cohort studies was evaluated using the Quality in Non- Randomised Studies (QUIN) tool. The overall risk judgments (Low, Moderate, or High) across seven bias domains are presented in Figure 2.

**Figure 3:**
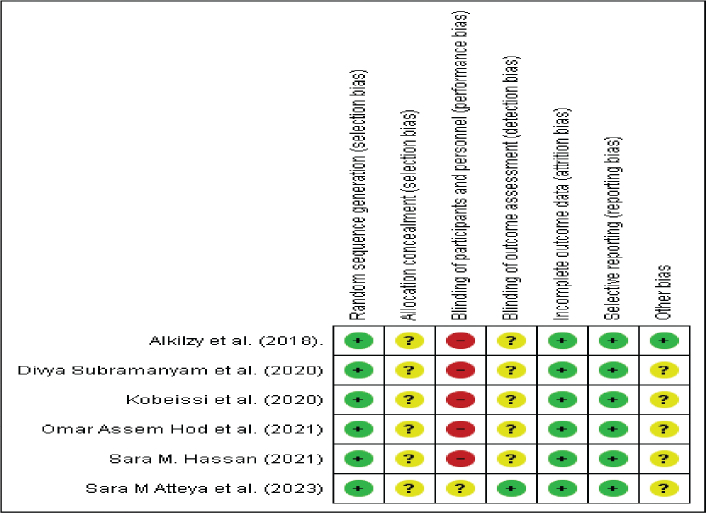
Risk of bias assessment for in vivo studies using Cochrane Risk of Bias 2.0 (RoB 2.0) tool. Source: Authors’ analysis.

For in vivo research (clinical trials), methodological quality assessment was performed according to the Cochrane Risk of Bias 2.0 (RoB 2.0) tool, as shown in Figure 3. This assessment covered five domains: the randomising process, deviations from intended interventions, missing outcome data, outcome assessment, and selection of reported outcomes. Each study was categorised as having low risk, some concerns, or high risk of bias, depending on the transparency and rigour of its reported methods.

**Figure 4:**
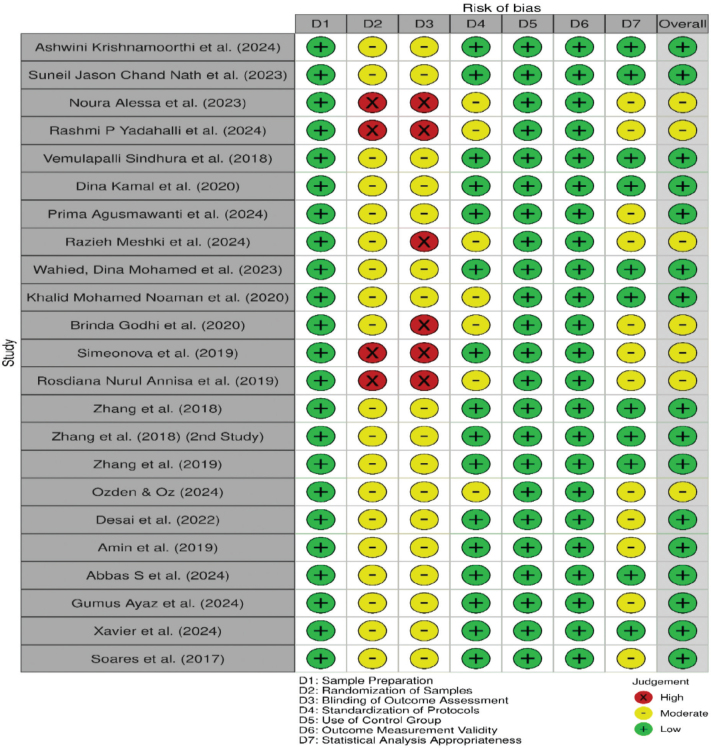
Risk of bias assessment for in vitro studies using a modified ROB-based assessment tool. Source: Authors’ analysis.

For in vitro studies, a structured and modified ROB-based assessment tool was used to evaluate seven criteria: sample size determination, randomisation, standardisation of procedures, blinding of outcome assessment, repetition of experiments, appropriate statistical analysis, and use of control groups. Each study was assessed and classified into low, moderate, or high-risk categories as illustrated in Figure 4. The detailed search strategy is provided in Appendix A.

## Results

Findings are presented descriptively, with relative improvements reported where available. A total of 31 studies were included, consisting of 8 in vivo clinical studies and 23 in vitro studies. The majority of included studies reported favourable remineralisation outcomes with emerging biocompatible materials compared to controls. A total of 77 full-text articles were excluded for the following reasons: lack of focus on emerging biocompatible materials (*n* = 25), use of fluoride-based biocompatible agents (*n* = 30), and absence of outcomes related to dental caries or remineralisation (*n* = 22), as shown in the PRISMA flow diagram (Figure 1). Detailed characteristics of the included studies are summarised in Table 1 (in vivo studies) and Table 2 (in vitro studies).

**Table 1:** Data extraction for in vivo studies.

S. No	Author (year)	Study design	Specimens used	Follow-up duration	Materials used	Mode of application	Outcome measures used	Clinical effectiveness	Patient-reported outcomes
1	Sara M Atteya et al. (2023) [8]	RCT (split-mouth)	Permanent teeth, non-cavitated carious lesions	6 months	SAP P11–4 + Nano Silver Fluoride vs SAP alone	Topical application	DIAGNOdent, ICDAS, morphometric	SAP + NSF showed higher remineralisation than SAP alone	Not reported
2	Divya Subramanyam et al. (2020) [9]	RCT	Primary molars	6 months	Aloe vera gel vs Formocresol	Direct pulp therapy (pulpotomy)	Clinical signs, radiographic evaluation	Comparable clinical success rates between Aloe vera and FC	Not directly assessed
3	Omar Assem Hod et al. (2021) [10]	RCT	Primary molars	6 months	Chitosan-modified GIC vs conventional GIC	Restorative filling	USPHS criteria	No significant difference between groups	Not reported
4	Schlee et al. (2018) [11]	Case series	Permanent teeth (proximal lesions)	12 months	SAP P11–4	Topical, direct application into lesion	Radiographic regression, subtraction analysis	Lesion depth reduced, early arrest observed	Not reported
5	Alkilzy et al. (2018) [12]	RCT	Permanent anterior teeth	6 months	SAP P11–4 + Fluoride varnish	Topical application	ICDAS scoring, DIAGNOdent	Significant remineralisation in SAP group	Not reported
6	Kobeissi et al. (2020) [13]	RCT	Children with WSLs	6 months	SAP P11–4 vs Tricalcium phosphate fluoride varnish	Topical application	DIAGNOdent, WSL index	SAP had comparable or slightly better outcomes	Not reported
7	Kind et al. (2017) [14]	Cohort Study	Permanent teeth with early caries	12 months	Self-assembling peptide	Topical application into lesion	Visual scoring, QLF	Significant improvement in enamel appearance and lesion regression	Not reported
8	Sara M. Hassan (2021) [15]	RCT	Permanent teeth with WSLs	6 months	Ginger & Rosemary Extract vs Fluoride Varnish	Topical gel	DIAGNOdent, WSL index	Herbal group showed comparable remineralisation to fluoride	Not reported

SAP: Self-assembling peptide; GIC: glass ionomer cement; RCT: Randomized Controlled Trial; NSF: Nano Silver Fluoride; USPHS: United States Public Health Service; FC: Formocresol; ICDAS: International Caries Detection and Assessment System; WSL: White Spot Lesion; QLF: Quantitative Light-Induced Fluorescence; CSP: Calcium Sucrose Phosphate; CPP: Casein Phosphopeptide; ACP: Amorphous Calcium Phosphate; ACPF: Amorphous Calcium Phosphate Fluoride; VHN: Vickers Hardness Number; SEM: Scanning Electron Microscopy; LRAP: Leucine-Rich Amelogenin Peptide; NaF: Sodium Fluoride; CT: Computed Tomography; CS: Chitosan; EDX: Energy Dispersive X-ray Analysis; VMT: Vickers Microhardness Test; SDF: Silver Diamine Fluoride; NHA: Nano Hydroxyapatite; TCP: Tricalcium Phosphate; CMC: Carboxymethyl Chitosan; TEM: Transmission Electron Microscopy; PAA: Polyacrylic Acid; BG: Bioactive Glass; APF: Acidulated Phosphate Fluoride; GSE: Grape Seed Extract; CESP: Chicken Eggshell Powder; SMH: Surface Microhardness; BAG: Bioactive Glass; PEO: Population Exposure Outcome; ROB: Risk of Bias; PRISMA: Preferred Reporting Items for Systematic Reviews and Meta-Analyses

**Table 2:** Data extraction for in vitro studies.

S. No	Author (year)	Study design	Specimens used	Follow-up duration	Materials used	Mode of application	Outcome measures used	Remineralisation outcome	Results/conclusion
1	Ashwini Krishnamoorthi et al. (2024) [16]	In vitro	Primary molars	21 days	P11–4, CSP, Bioactive glass, CPP-ACP, CPP-ACPF	Topical application (micropipette)	Surface Vickers microhardness (VHN), SEM	All P11–4 combinations showed increased surface microhardness and improved enamel morphology under SEM; highest effectiveness observed in P11–4 + CPP-ACPF.	P11–4 combined with calcium phosphate-based agents led to significant remineralisation; P11–4 + CPP-ACPF group showed the highest efficacy.
2	Suneil Jason Chand Nath et al. (2023) [17]	In vitro	Premolars	8 days	P11–4, P26, LRAP, 5% NaF, DI water	Topical application (P11–4 via sponge; others in solution)	Micro-CT (MD), Nano indentation (H, EM)	P11–4 and P26 significantly increased mineral density, hardness, and elasticity modulus compared to NaF and LRAP, especially at deeper enamel layers (60 µm).	P11–4 & P26 outperformed NaF & LRAP at 60 µm depth; SAPs show strong remineralisation potential.
3	Noura Alessa et al. (2023) [18]	In vitro	Premolars	12 days	Bioactive glass, P11–4, Ozone remineralising agents, Deionised water (Control group)	Topical application (ozone via ozone unit & others in solution)	Vickers microhardness (VHN)	Increased microhardness post-remineralisation.	Bioactive glass and self-assembling peptides showed higher remineralising capacity.
4	Rashmi P Yadahalli et al. (2024) [19]	In vitro	Premolars	14 days	Bioactive glass, P11 4, Arginine bicarbonate remineralising agents, deionised water(control)	Topical application (in solution)	Vickers microhardness (VHN)	Increased microhardness post-remineralisation.	Bioactive glass and self-assembling peptides showed higher remineralising capacity.
5	Vemulapalli Sindhura et al. (2018) [20]	In vitro	Premolars	1 week, 1 month, 3 months	SAP P11 and CPP ACP	Topical (SAP via brush after etching; CPP-ACP via gloved finger)	SEM, EDX, Ca/P Ratio	P11–4 showed gradual increase in Ca/P ratio over time; CPP-ACP showed early spike then decline.	At 3 months, SAP P11–4 showed higher Ca/P ratio and better mineral deposition than CPP-ACP.
6	Dina Kamal et al. (2020) [21]	In vitro	Molars	1 week, 4 weeks	Fluoride varnish, CPP-ACPF varnish, SAP P11–4, SAP + Fluoride, SAP + CPP-ACPF	Topical application	Surface Vickers microhardness (VHN), SEM	All groups showed remineralisation; combination groups (SAP + Fluoride and SAP + CPP-ACPF) showed higher microhardness values.	Combination of SAP with CPP-ACPF or Fluoride showed superior and faster remineralisation; synergistic effect evident from 1 to 4 weeks.
7	Prima Agusmawanti et al. (2024) [22]	In vitro	Mandibular incisors	7 days	QP3VH, NaF, QP3VH + NaF, QP3VH + CS, and saline distilled water	Topical application (in solution)	SEM, EDX, Ca/P Ratio	QP3VH-based groups showed notable enamel surface improvement; combination with chitosan and/or NaF further enhanced remineralisation.	QP3VH peptide significantly improved remineralisation; synergistic effect with NaF or chitosan shown by smoother enamel and higher Ca/P ratio.
8	Razieh Meshki et al. (2024) [23]	In vitro	Primary canine	7 days	Bioglass-Chitosan solution, Chitosan, Bioglass solution, SDF, Remineralisation solution, Distilled water	Topical application (except SDF, applied once)	Vickers microhardness (VHN) Test (VMT)	Increased microhardness post-remineralisation.	Bioglass-chitosan solution showed the highest improvement in Vickers microhardness (VHN); chitosan and bioglass also effective.
9	Wahied, Dina Mohamed et al. (2023) [24]	In vitro	Permanent molars	42 hours	Nano β-TCP, Nβ-TCP + Chitosan, NHA, NHA + Chitosan	Topical application (Gel applied with micro-brush)	Vickers microhardness (VHN) Number (VHN), SEM imaging	All test groups showed increased microhardness; NHA + Chitosan most effective.	NHA + Chitosan group exhibited 55.10% Vickers microhardness (VHN) increase; chitosan enhanced both NHA and Nβ-TCP efficacy.
10	Khalid Mohamed Noaman et al. (2020) [25]	In vitro	Premolars with subclinical lesions	3 and 6 months	Phosphorylated Chitosan + ACP, CPP-ACP, Fluoride	Topical brushing	DIAGNOdent, EDAX (Ca/P ratio)	Chitosan group showed highest remineralisation; time-dependent increase.	Chitosan significantly more effective at 6 months; fluoride lowest remineralisation.
11	Brinda Godhi et al. (2020) [26]	In vitro	Premolars	21 days	Chitosan (2.5 mg/ml), Ginger-Manuka honey mix	Topical application	QLF parameters: ΔF, ΔF Max, ΔQ	Chitosan showed significant remineralisation; Ginger-Honey moderate.	Chitosan highest potential; Ginger-Honey moderate from Day 14 onward.
12	Simeonova et al. (2019) [27]	In vitro	Permanent molars and premolars	42 hours (6 h × 7 sessions)	Hybrid chitosan/calcium phosphates microgels	Immersion for 6 h per cycle	SEM, ATR-IR spectroscopy	Formation of enamel-like nanocrystalline structures.	Chi_CaP MGs effectively promoted remineralisation, forming stable enamel-like phases.
13	Rosdiana Nurul Annisa et al. (2019) [28]	In vitro	Dentin collagen samples from human teeth	Not explicitly stated	Carboxymethyl Chitosan + Amorphous Calcium Phosphate (CMC/ACP)	Applied as cavity base; stored in PBS at 37°C	EDX, TEM	Increased Ca and P levels; intra- and extrafibrillar mineral deposition.	CMC/ACP showed superior dentin remineralisation and mineral content restoration.
14	Zhang et al. (2018) [29]	In vitro	Molars	7 days	Bioactive glass (BG), BG + PAA, Chitosan pre-treatment (CS-BG, CS-BG + PAA), remineralising solution (RS), deionised water	Topical application	Raman spectroscopy, Knoop surface & cross-sectional Vickers microhardness (VHN), SEM	CS-BG group showed greatest surface and subsurface remineralisation; CS-BG + PAA had larger enamel-like crystals.	Chitosan enhanced subsurface remineralisation even in presence of pellicle; potential for lesion consolidation.
15	Zhang et al. (2018) [30]	In vitro	Molars	7 days	Bioglass (BG), Bioglass + PAA, Chitosan + BG, Chitosan + BG + PAA, Standard remineralising solution, Deionised water	Topical application	Raman spectroscopy, ATR-FTIR, Knoop Vickers microhardness (VHN), SEM, EDX	CS-BG + PAA group had greatest subsurface mineral content and hardness recovery.	Chitosan pre-treatment significantly improved bioglass remineralisation; supports chitosan-bioglass use for subsurface lesions.
16	Zhang et al. (2019) [31]	In vitro	Molars	7 days	Chitosan-bioglass complex (CBS), Bioglass with chitosan pre-treatment (CB), standard remineralisation solution (RS), deionised water (NC)	Topical application after salivary pellicle	Raman mapping, Knoop Vickers microhardness (VHN), SEM, EDX	CBS showed greatest subsurface mineral deposition and hardness recovery.	CBS significantly promoted remineralisation with dense HA-like mineral formation; effective even with pellicle.
17	Ozden & Oz (2024) [32]	In vitro	Mandibular molars	14 days	Grape Seed Extract (25%), CPP-ACP, APF (alone and combinations)	Topical (cotton applicator 15 seconds, held 1 minutes)	Surface Vickers microhardness (VHN), SEM	All groups showed some remineralisation; GSE alone less effective than when combined.	GSE enhanced remineralisation when combined with CPP-ACP or APF; significant hardness increase in combined groups.
18	Desai et al. (2022) [33]	In vitro	Primary molars	10 days	CPP-ACPF, TCP, Grape Seed Extract, Deionised Water	Topical application on enamel window	Vickers microhardness (VHN), SEM-EDX, CBCT	Grape seed extract showed highest remineralisation.	Grape seed extract significantly enhanced Vickers microhardness (VHN) and mineral gain; superior to CPP-ACPF and TCP.
19	Amin et al. (2019) [34]	In vitro	Primary anterior teeth	8 days	Grape Seed Extract (GSE), Sodium Fluoride (NaF, 1000 ppm)	Topical application	Vickers microhardness (VHN), SEM-EDX	GSE promoted mineral deposition but had lower microhardness than NaF.	GSE showed remineralisation potential but less effective than NaF; suitable natural alternative.
20	Abbas S et al. (2024) [35]	In vitro	Upper first premolars	14 days	CPP-ACP (control), GSE, Chicken Eggshell Extract (CESP), GSE + CESP	Topical application	Vickers microhardness (VHN) (SMH)	GSE + CESP group showed highest SMH values; CESP alone also highly effective.	Combination of GSE and CESP demonstrated synergistic effect; significant enamel remineralisation in all groups.
21	Gumus Ayaz et al. (2024) [36]	In vitro	Premolars	6 days	CPP-ACP, Rosemary Oil (RO), Ginger-Honey, Ginger-Honey–Cocoa, Grape Seed Extract (GSE), Control Group	Topical application (using applicator)	Vickers microhardness (VHN), DIAGNOdent	GSE showed the highest remineralisation potential among tested agents.	GSE most effective; herbal-based tooth creams non-invasive and suitable for early caries repair.
22	Xavier et al. (2024) [37]	In vitro	Premolars	4 weeks	CPP-ACPF, Tricalcium phosphate with fluoride (TCP-F), Calcium sucrose phosphate (CSP), P11–4	Topical application	Surface Vickers microhardness (VHN), SEM, EDX	P11–4 showed highest VHN; TCP-F and P11–4 had greater mineral gain (EDX); CSP moderate; CPP-ACPF least effective.	P11–4 exhibited greatest remineralisation; TCP-F also strong performer.
23	Soares et al. (2017) [38]	In vitro	Premolars	30 days	CPP-ACPF, TCP-F, CSP, P11–4	Topical application	Surface Vickers microhardness (VHN), SEM	P11–4 showed highest microhardness recovery (62.06%), followed by CPP-ACPF, BAG, and HA gel.	P11–4 highest remineralising capacity; SEM confirmed organised mineral deposition.

SAP: Self-assembling peptide.

### In vivo (clinical) evidence

Eight in vivo studies evaluated the clinical performance of emerging biocompatible materials, primarily self-assembling peptides (SAP P11–4), Aloe vera, chitosan-modified glass ionomer cement (GIC), and selected herbal-derived agents. The most frequently assessed clinical parameters included lesion regression, surface integrity, remineralisation potential, and pulpotomy success, evaluated using tools such as ICDAS scoring, DIAGNOdent, radiographic imaging, and USPHS criteria.

Self-assembling peptide SAP P11–4 demonstrated favourable remineralisation outcomes in seven out of eight clinical studies, particularly when used alone or in combination with fluoride or calcium phosphate–based agents. Aloe vera exhibited comparable clinical success to formocresol when used as a pulpotomy medicament in primary teeth. Chitosan-modified GICs showed clinical performance similar to conventional GICs, with no significant differences in restoration integrity or short-term outcomes.

However, most clinical studies were characterised by limited sample sizes and short follow-up durations. Importantly, none of the included in vivo studies reported validated patient-reported outcome measures, such as patient comfort, satisfaction, or oral health–related quality of life.

### In vitro evidence

Twenty-three in vitro studies investigated the remineralisation potential of biocompatible materials, including SAP P11–4, chitosan-based formulations, bioactive glass, grape seed extract, and herbal derivatives such as ginger and rosemary. Laboratory assessments predominantly employed surface microhardness testing (Vickers or Knoop), scanning electron microscopy with energy-dispersive X-ray analysis (SEM/EDX), and calcium- to-phosphorus (Ca/P) ratio analysis.

Across the majority of laboratory studies, both SAP P11–4 and chitosan-based materials demonstrated greater remineralisation potential compared to control groups, reflected by increased surface hardness, improved enamel morphology, and enhanced mineral deposition. Several studies reported additive or synergistic effects when SAP P11–4 was combined with CPP-ACPF or when chitosan was combined with bioactive glass or nano-hydroxyapatite.

Most in vitro studies were assessed as having a moderate risk of bias, primarily due to the absence of sample size calculations, lack of randomisation, and absence of blinding during outcome assessment. In contrast, the majority of in vivo studies demonstrated low to moderate risk of bias, with no study classified as high risk. These laboratory findings represent surrogate indicators of remineralisation and should not be directly extrapolated to clinical effectiveness.

## Discussion

This systematic review found that emerging biocompatible materials, particularly SAP P11–4 and chitosan-based formulations, consistently demonstrated remineralisation potential, with evidence derived from both in vitro investigations and a limited number of clinical studies. While available clinical evidence supports the use of self-assembling peptides in the management of early, non-cavitated carious lesions, the majority of evidence for herbal-derived materials remains laboratory-based, underscoring the need for further well-designed clinical validation [8, 12, 14]

The growing interest in biocompatible materials within restorative dentistry, especially those promoting biomimetic remineralisation, is reflected in the findings of this review. Among the evaluated agents, SAP P11–4 is the most extensively studied, with both laboratory and clinical investigations indicating its ability to facilitate enamel repair by forming a biomimetic scaffold that supports hydroxyapatite deposition within early carious lesions [12, 14, 17]. This mechanism closely resembles natural remineralisation pathways and aligns with minimally invasive caries management principles [12, 13]. Clinical studies suggest that SAP P11–4 may contribute to lesion stabilisation and hardness improvement; however, these findings are based on relatively short follow-up periods and modest sample sizes.

Chitosan-based materials exhibited antimicrobial and remineralisation-supportive properties, particularly when combined with bioactive glass or hydroxyapatite [10, 23, 29]. Laboratory findings indicate that such combinations may enhance mineral deposition and enamel surface integrity. Nevertheless, clinical evidence evaluating chitosan-modified restorative materials remains limited, and conclusions regarding long-term clinical effectiveness should therefore be interpreted with caution [23, 29].

Herbal-derived agents, including grape seed extract, Aloe vera, and combined herbal formulations (such as ginger, rosemary, and honey), demonstrated potential remineralisation and antimicrobial effects primarily in in vitro models [32, 33]. Improvements in enamel hardness and mineral content were consistently reported under laboratory conditions; however, clinical data supporting their effectiveness in patient care are sparse [32, 33]. As a result, current evidence for herbal agents should be considered preliminary [9, 15].

The moderate risk of bias observed across most in vitro studies, primarily due to the absence of randomisation, blinding, and sample size calculation, may have inflated reported effect sizes. These methodological limitations reduce confidence in direct clinical translation and highlight the need for standardised in vitro protocols and well-designed confirmatory clinical trials (Figure 4).

### Emerging green and nano-enabled biomaterials

Recent advances in restorative dentistry have increasingly focused on green synthesis approaches and nano-enabled biomaterials to enhance antimicrobial activity, remineralisation efficacy, and mechanical performance while improving sustainability and biocompatibility. Plant-derived bioactive compounds synthesised via green routes have demonstrated improved antibacterial effects against cariogenic microorganisms, along with enhanced mineral deposition when incorporated into restorative or remineralising systems. In addition, nanoparticle-mediated modifications, including bioactive glass, nano-hydroxyapatite, and metal or polymer-based nanoparticles, have been shown to improve surface hardness, interfacial bonding, and resistance to degradation of restorative materials. Natural resin-based products such as propolis have also been explored as modifiers of GICs, demonstrating enhanced antimicrobial activity and acceptable physico-mechanical properties in laboratory and systematic review evidence. These strategies offer the dual advantage of reducing reliance on chemically intensive manufacturing processes while improving functional performance. Although the majority of evidence supporting these approaches remains laboratory-based, emerging findings suggest that green-synthesised and nano-enhanced biomaterials may represent promising adjuncts for future restorative applications, warranting further clinical investigation [39–44].

Although these materials are generally regarded as safe, biocompatible and cost-effective, their clinical applicability cannot yet be considered equivalent to established fluoride-based therapies. Collectively, the findings suggest a shift towards more biologically compatible and minimally invasive strategies in restorative dentistry. In situations where fluoride use is limited or contraindicated, these agents may represent potential adjunctive or alternative approaches, pending confirmation through robust, long-term randomised clinical trials [12, 15].

### Public health significance

Dental caries is a major global health issue that affects billions of people and has a big effect on their quality of life. Fluoride has been the most important part of preventive programmes for almost 50 years. Concerns about fluorosis and toxicity at high exposure levels have led us to look further for safer, more sustainable alternatives. Biocompatible remineralising agents may provide such an opportunity. This is because their integration into community and clinical practice may reduce the global prevalence of untreated caries and improve access to safe and minimally invasive treatments [2, 6].

### Clinical application

From a clinical point of view, all of the materials reviewed here (Tables 1 and 2) are promising in preventive as well as restorative care. Self-assembling peptides, especially SAP P11–4, seem to be good for treating early non-cavitated lesions and white spot lesions because they imitate natural mineralisation pathway and offer a technique to repair enamel that is not too invasive [8, 12, 13]. Using self-assembling peptide P11–4 alongside fluoride or calcium-phosphate–based agents may enhance treatment outcomes.

Chitosan has the potential to be used into restorative materials like GICs, varnishes, and even everyday dentifrices because of its dual antibacterial and remineralising qualities [10, 24, 29]. The use of herbal and plant-derived materials, such as grape seed extract, Aloe vera, and ginger-rosemary formulations, has also demonstrated promising results in pulp therapy and remineralisation [9, 15, 36]. This is particularly true in paediatric dentistry, where biocompatibility is of upmost importance.

These applications collectively underscore the adaptability of biocompatible agents in both preventive and restorative contexts, advocating for a transition towards more biologically compatible and patient-centric therapeutic methodologies.

## Limitations

There was significant variability in the design of studies, formulations of agents, treatment duration and duration of patient evaluation evidence. The predominant evidence was derived from in vitro studies with limited follow-up periods, restricting applicability to clinical settings. Patient-reported outcomes such as comfort, satisfaction, or quality of life were rarely assessed and therefore could not be meaningfully synthesised. Another limitation, that becomes apparent due to the methodological inconsistencies such as lack of randomisation, blinding, and standardised protocols results in moderate risk of bias. These constraints highlight the urgent need for well-structured, multicentre randomised controlled trials utilising standardised methods. Reporting bias was not assessed because a meta-analysis was not performed. The certainty of evidence was not formally graded due to narrative synthesis.

## Recommendations

We recommend conducting high-quality, long-term randomised trials with proper sample sizes and methods that validate the clinical effectiveness and durability of these biocompatible materials. The introduction of patient-centred outcomes, including comfort, satisfaction and quality of life would also help to discern real-world benefits farther down the road. Studies of synergistic combinations – for example, peptides in combinations with calcium phosphate or chitosan and bioglass may uncover maximum remineralisation efficacy. Analysing accessibility, safety, and cost-effectiveness among diverse populations will be crucial for practical implementation. In order to ensure that these promising materials can be translated into broadly accessible, scalable, and sustainable solutions for everyday dental care, researchers must prioritise the bridging of the divide between laboratory discoveries and practical implementations.

## Conclusion

This review shows that emerging biocompatible materials, particularly self-assembling peptides and chitosan-based formulations offer promising results in promoting enamel remineralisation and improving restorative outcomes. Findings from laboratory research consistently support their effectiveness, and early clinical evidence suggests they could serve as potential adjunctive or alternative approaches to fluoride-based materials. However, variations in study methods and the predominance of in vitro designs limit the strength of current evidence. Well-designed, long-term clinical trials are needed to confirm their effectiveness, durability, and patient-centred advantages in routine dental care.

## Data and materials availability

All data generated or analysed during this study are included in this published article and its supplementary information files. No additional datasets were generated.

## Appendices

### Appendix A – Search Strategy

#### PubMed

((”Dental Caries”[Mesh] OR dental caries OR enamel lesion* OR demineralization)

AND (remineralization OR remineralisation OR ”enamel repair” OR ”dentin repair”)

AND (”Biocompatible Materials”[Mesh] OR biocompatible OR biomaterial*

OR ”self assembling peptide” OR P11–4 OR chitosan

OR ”Aloe vera” OR ”grape seed” OR herbal OR ”non-fluoridated”))

Filters applied: English language, 2015–2025

#### Cochrane Library

(dental caries OR enamel lesion* OR demineralization)

AND (remineralization OR remineralisation OR enamel repair)

AND (”self assembling peptide” OR P11–4 OR chitosan

OR ”Aloe vera” OR ”grape seed” OR herbal OR biocompatible)

Limits: Trials, Reviews; 2015–2025; English

#### Scopus

(TITLE-ABS-KEY (caries OR ”white spot lesion*” OR demineralization)

AND TITLE-ABS-KEY (remineralization OR remineralisation)

AND TITLE-ABS-KEY (”self assembling peptide” OR P11–4 OR chitosan

OR ”grape seed” OR ”Aloe vera” OR herbal OR biocompatible

OR ”natural material”))

AND (LIMIT-TO (LANGUAGE, ’English’))

AND (PUBYEAR > 2014)

#### EBSCOhost

(dental caries OR enamel lesion* OR demineralization)

AND (remineralization OR enamel repair)

AND (”self assembling peptide” OR P11–4 OR chitosan

OR ”Aloe vera” OR ”grape seed” OR herbal OR biocompatible)

Limiters applied: 2015–2025; English language; Academic Journals
